# HIV-, HCV-, and Co-Infections and Associated Risk Factors among Drug Users in Southwestern China: A Township-Level Ecological Study Incorporating Spatial Regression

**DOI:** 10.1371/journal.pone.0093157

**Published:** 2014-03-31

**Authors:** Yi-Biao Zhou, Qi-Xing Wang, Song Liang, Yu-Han Gong, Mei-xia Yang, Shi-Jiao Nie, Lei Nan, Ai-Hui Yang, Qiang Liao, Yang Yang, Xiu-Xia Song, Qing-Wu Jiang

**Affiliations:** 1 Department of Epidemiology, School of Public Health, Fudan University, Shanghai, China; 2 Key Laboratory of Public Health Safety, Ministry of Education, Fudan University, Shanghai, China; 3 Tropical Disease Research Center, Fudan University, Shanghai, China; 4 Center for Disease Prevention and Control of Liangshan Prefecture, Sichuan, China; 5 Department of Environmental and Global Health, College of Public Health and Health Professions, University of Florida, Gainesville, Florida, United States of America; 6 Emerging Pathogens Institute, University of Florida, Gainesville, Florida, United States of America; 7 Xuhui Center for Disease Prevention and Control, Shanghai, China; 8 Department of Biostatistics, University of Florida, Gainesville, Florida, United States of America; Temple University School of Medicine, United States of America

## Abstract

**Background:**

The human immunodeficiency virus (HIV) and hepatitis C virus (HCV) are major public health problems. Many studies have been performed to investigate the association between demographic and behavioral factors and HIV or HCV infection. However, some of the results of these studies have been in conflict.

**Methodology/Principal Findings:**

The data of all entrants in the 11 national methadone clinics in the Yi Autonomous Prefecture from March 2004 to December 2012 were collected from the national database. Several spatial regression models were used to analyze specific community characteristics associated with the prevalence of HIV and HCV infection at the township level. The study enrolled 6,417 adult patients. The prevalence of HIV infection, HCV infection and co-infection was 25.4%, 30.9%, and 11.0%, respectively. Prevalence exhibited stark geographical variations in the area studied. The four regression models showed Yi ethnicity to be associated with both the prevalence of HIV and of HIV/HCV co-infection. The male drug users in some northwestern counties had greater odds of being infected with HIV than female drug users, but the opposite was observed in some eastern counties. The ‘being in drug rehabilitation variable was found to be positively associated with prevalence of HCV infection in some southern townships, however, it was found to be negatively associated with it in some northern townships.

**Conclusions/Significance:**

The spatial modeling creates better representations of data such that public health interventions must focus on areas with high frequency of HIV/HCV to prevent further transmission of both HIV and HCV.

## Introduction

Human immunodeficiency virus (HIV) and hepatitis C virus (HCV) have become a rapidly emerging global public health problem. An estimated 34 million persons currently live with HIV/AIDS, and 170 million people may be infected with HCV [1]–[3]. Co-infection with HIV and HCV is also very common in certain populations, especially in intravenous drug users. This is because HIV and HCV share similar routes of transmission, including blood-blood contact, such as occurs during intravenous drug use, and sexual contact [1]–[6]. It has been estimated that, in the United States, 25% of people infected with HIV are also infected with HCV. The prevalence of co-infection with HIV and HCV can surpass 90% among IDUs [4], [7], [8]. HIV and HCV infections, like most other infectious diseases, are known to cluster in relation to risk factors, especially substantial risk factors like needle sharing and migration [9]–[12]. Many studies have been performed to investigate the association between demographic and behavioral factors and HIV or HCV infection among drug users at the individual level with contradictory results [13], [14]. For this reason, it is important to recognize that many of these studies may be affected by regional differences and methodological limitations that may undermine the reliability and stability of their results. A systematic review and meta-analysis showed that gender was not associated with HIV. However, after stratifying by geographical location, the odds of male drug users being infected by HIV were found to be higher than among female drug users in high-transmission areas. However, the opposite was true in low-transmission areas [13]. Known risk factors like intravenous drug use and sharing needles were found to be associated with HIV infection at the individual level in logistic regression, but they were not found to be associated with the prevalence of HIV at the population level [15]. Coefficients and standard error of regression analyses may be overestimated due to potential spatial autocorrelation in data like that concerning HIV infection and needle sharing (i.e., the data may be not independent) [16]. At present, most studies on risk factors of HIV and HCV infection focus mainly on the individual level. Few studies have used spatial regression techniques to analyze risk factors of HIV or HCV infection at the population level [13], [15]. For this reason, the present study aims to identify some community characteristics associated with the prevalence of HIV and HCV infection among drug users in an Yi Autonomous Prefecture using spatial regression techniques. Studying risk factors associated with the prevalence of HIV and HCV infection at the township level may provide a fresh look into the risk factors of these infections and informed insights into what drives these infections in this part of the globe.

## Materials and Methods

### Study setting and population

Liangshan Yi Autonomous Prefecture is in Sichuan Province. It is located in southwestern China. HIV/AIDS is endemic there. It has a population of 4.873 million people in 618 townships and 16 counties and a city. Approximately 50% of the population belongs to the Yi ethnic group. One of the first eight national methadone clinics was established in this prefecture in 2004 [17]. As of the end of 2012, there were 10 fixed methadone clinics and 1 mobile methadone clinic. All patients attending the 11 methadone clinics from March 2004 to December 2012 were selected as the study population. Details on these national methadone patients have been reported previously [17]–[19]. All these national methadone patients were tested for both HIV and HCV infections one month after entry into the methadone clinics. Ethical approval was granted by the institutional review board of the National Center for AIDs/STD Control and Prevention, China CDC, and written informed consent was obtained from all participants.

### Data collection

This study was approved by the Center for Disease Prevention and Control of Liangshan Prefecture. Permission was issued to analyze data collected from 2004 to 2012 from the Liangshan Prefecture. These data were stored in the national methadone database. Information regarding the demographic and clinical characteristics and drug use behaviors of all entrants to the 11 methadone clinics in the Yi prefecture from March 2004 to December 2012 were collected from the national methadone database [13], [14], [18]. Data regarding HIV/HCV infections, gender, age, ethnicity, drug use behaviors and residency of the methadone patients were used in the present study. Data were entered into Microsoft Excel version 2010 (Microsoft Corp., Redmond, WA, U.S.) and the Statistical Package for Social Sciences (SPSS Inc., Chicago, IL, 2007). Individuals were considered infected with HIV or HCV if they had at least one positive set of test results.

Under the Chinese government, the smallest administrative unit is the village, and the next smallest unit is the township. However, no geographical data on village zones are released to the public; so, the township is the basic geographical unit of the present analysis. The neighborhood is defined as the township in the present study.

Dependent variables: the prevalence of HIV infection of a township containing drug users who attended the methadone clinics was defined as the proportion of patients infected with HIV. The prevalence of HCV infection and of HIV/HCV co-infection was calculated in a similar fashion. In order to reduce variance instability of the prevalence of infection, the variance instability was adjusted using the spatial empirical Bayes (EB) smoother, here referred to as the local EB smoother [20]–[23]. This spatial EB smoother uses Bayesian principles to guide the adjustment of a rate estimate by taking into account information in the limited subsample (allowing for greater flexibility in modeling heterogeneity). This information is defined by a spatial weight. The principle is referred to as shrinkage. I.e. if a prevalence estimate has a small variance (e.g., is based on a large, at-risk population), then it will remain essentially unchanged. In contrast, if a rate has a large variance (e.g., based on a small, at-risk population), then it will shrink towards the local mean [20], [21]. For this reason, these three adjusted dependent variables (spatial EB adjusted prevalence of HIV infection, spatial EB adjusted prevalence of HCV infection, and spatial EB adjusted prevalence of HIV/HCV co-infection) were used in this study.

Independent variables: The relative number of men was defined as the ratio of men living in each township to total population. Like the percentage of male participants, the percentage of the methadone patients in each of four age groups (<30, 30–39, 40–49, and ≥50 years) and in three ethnic groups (Yi, Han, and other) were calculated. The relative number of individuals who engaged in intravenous drug use, the sharing of syringes, and drug rehabilitation were also calculated. All the percentages were adjusted using a spatial (EB) smoother.

### Statistical analyses

First, standard ordinary least squares (OLS) linear regressions were fitted to estimate the association of the independent variables and the dependent variables using the backward method (a variable selection procedure in which all variables are entered into the equation and then sequentially removed). Preliminary data analyses showed the prevalence of HIV, HCV, and co-infection were skewed, so a square root transformation of these dependent variables was performed. The age groups were compared to each other (i.e., <30 years vs. all other groups, 30–39 years vs. all other groups, 40–49 years vs. all other groups, and ≥50 years vs. all other groups). Ethnic groups were compared similarly. A tolerance test was performed to determine whether the independent variables were correlated in a multicollinear fashion.

Second, three spatial regression models were fitted to examine the relationships between the dependent variables and independent variables: spatial lag model (SLM), spatial error model (SEM), and geographically weighted regression (GWR) model. In order to control and prevent multicollinearity, only variables found to be statistically significant in the OLS regression model were used in these three spatial models. Spatial regression methods allow spatial dependency to be taken into consideration, preventing some statistic problems (e.g., unstable parameters and unreliable significance tests). They also provide some information regarding spatial relationships among the variables involved [24]. SLM directly incorporates spatial autocorrelation into the model by including a spatial lag term (*ρ*). SEM works similarly to SLM, except the spatial autocorrelation term applies to the error terms (λ) of the neighboring townships rather than their dependent variable values [25]. SLM and SEM were estimated using the generalized method of moments in GeoDaSpace (alpha) revision 0.8.1 (https://geodacenter.asu.edu/software/downloads/geodaspace). In this way, the two models implemented in GeoDaSpace were able to control for both spatial autocorrelation and heteroskedasticity [26]–[29]. The spatial weight of both SLM and SEM was based on the first-order Queen's contiguity rule (i.e. if two townships shared a boundary or node, the weight was equal to 1; otherwise it was zero). GWR is a new local modeling technique used for analysis of spatially varying relationships. This method allows local (as opposed to global) models of relationships to be measured and mapped. GWR was estimated with GWR 4.0 software (http://www.st-andrews.ac.uk/geoinformatics/gwr/gwr-downloads/), and “Gaussian,” “Adaptive Gaussian,” “Golden section search,” and “AICc” served as “Model type,” “Geographic kernel,” “Method of optimal bandwidth search,” and “Criterion for optimal bandwidth,” respectively.

## Results

### HIV infection and associated risk factors

**Figure 1:**
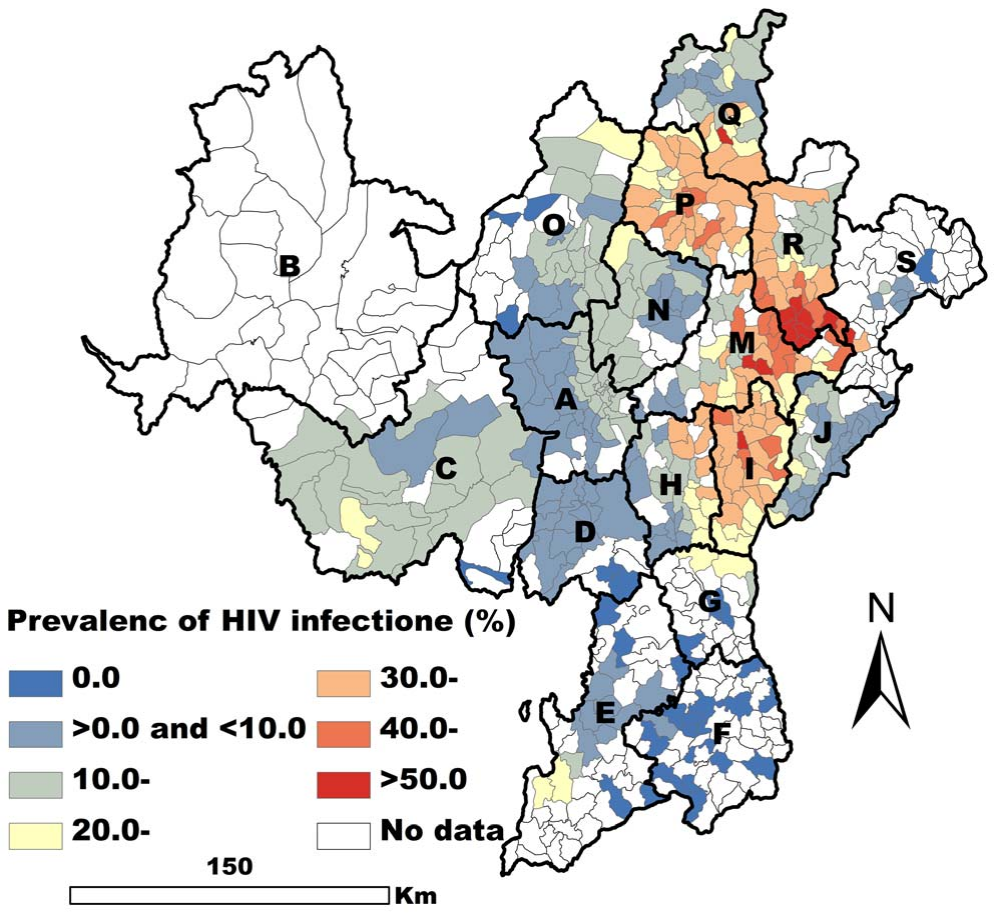
Spatial EB smoothed map of the prevalence of HIV infection by township.

A total of 6417 methadone patients with complete information regarding address, risk factors, and results of HIV tests were obtained from the database. The search period covered the 11 methadone clinics from March 2004 through the end of 2012. 25.4% (1628/6417) of these patients were HIV-positive. These methadone patients came from the 389 of the 618 townships of the Yi prefecture (Figure 1).

In the OLS regression model, there were five variables that were closely related to the prevalence of HIV infection (*P*<0.001). Of these five variables, drug rehabilitation was negatively associated with HIV infection, and the other four variables were positively associated with HIV infection. The fit of the OLS model was good (Akaike info criterion (AIC)  = −616.55), and the tolerance values indicated slight problem with multicollinearity (The multicollinearity condition number was 19.12). However, the low probabilities of Jarque-Bera test scores and the White test scores indicated non-normal distribution of the error and existence of heteroskedasticity. Moran's I (error) score was positive and highly significant (*P*<0.001) indicating strong positive spatial autocorrelation of the residuals (Table 1).

**Table 1:** Association of the prevalence of HIV infection and risk factors.

	OLS				SLM				SEM			
Model fit												
R^2^	0.6763				0.8433				0.6457			
Adjusted R^2^	0.6720				0.6994				-			

Model estimation	Coefficient	t-statistic		*P*	Coefficient	Z-value		*P*	Coefficient	Z-value		*P*
Constant	0.0329	1.0671		0.2866	0.0138	0.6380		0.5234	0.0209	0.8732		0.3826
Rate of intravenous drug use	0.6267	12.1136		0.0000	0.3277	5.9311		0.0000	0.4558	6.5209		0.0000
Rate of sharing syringes	0.6850	15.3343		0.0000	0.3658	6.7158		0.0000	0.6070	9.3091		0.0000
Rate of participation in drug rehabilitation programs	−0.3433	−8.0777		0.0000	−0.1830	−4.9287		0.0000	−0.3306	−5.6952		0.0000
Relative number of Yi	0.3227	15.6781		0.0000	0.1511	5.3974		0.0000	0.1966	7.1490		0.0000
Relative number of men	−0.1258	−2.7398		0.0064	−0.0796	−2.4412		0.0146	0.0900	1.6387		0.1013
*ρ*/λ*	-	-		-	0.5455	7.1347		0.0000	0.6869	27.1175		0.0000

Diagnostic tests	Value		*P*		Value		*P*		Value		*P*	
Normality of errors												
Jarque-Bera test	4.092		0.1292						-		-	
Heteroskedasticity												
White test	193.109		0.0000		-		-		-		-	
Spatial dependence												
Moran's I (error)	16.257		0.0000		-		-		-		-	
Anselin-Kelejian Test	-		-		1.8691		0.1716		-		-	

*ρ: spatial lag term; λ: the error terms.

The results of the SLM regression model changed slightly (Table 1). The absolute values of the regression coefficients of all variables were lower than in the OLS regression model, and the ranking of the regression coefficients was the same in the SLM and OLS regression models (Table 1). The coefficient parameter (*ρ*) of the spatial lag term was closely related to township-specific prevalence of HIV infection (*ρ* = 0.55, *P*<0.001), indicating the spatial dependence inherent in the prevalence of HIV infection, i.e. high prevalence of HIV infection was associated with high prevalence of infection in nearby townships (Table 1). The fit of the SLM regression model was much better than that of the OLS regression model, as indicated by higher values of R-squared and the absence of spatial dependence.

The results of the SEM regression model also changed slightly, especially for the male variable, and the regression coefficient of the male variable ceased to achieve statistical significance (*P*>0.05) (Table 1). The absolute values of the regression coefficients of all variables were lower than in the OLS regression model (Table 1).

**Figure 2:**
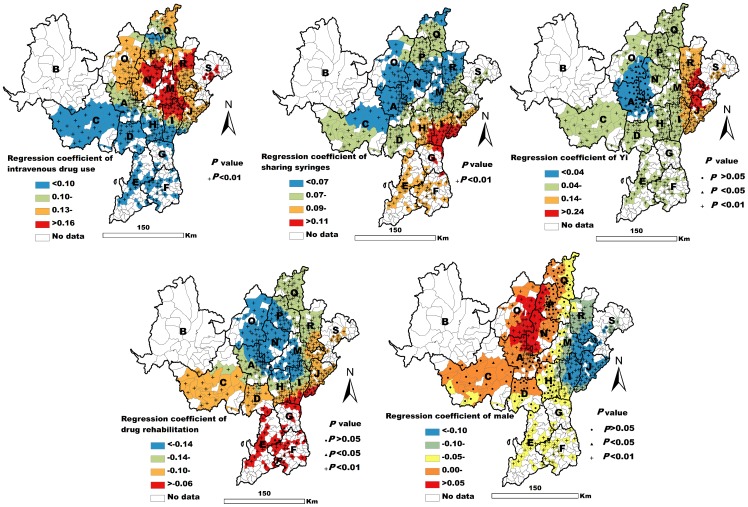
Spatial map of the GWR coefficients of five variables associated with HIV infection.

The fit of the GWR model was very high (AIC = −820.11, R^2^ = 0.82, adjusted R^2^ = 0.81). The GWR results showed both intravenous drug use and sharing syringes to be significantly related to the prevalence of HIV infection in all observed townships (*P*<0.01). However, the values of these GWR standardization coefficients were different in different townships (Figure 2). The Yi ethnicity variable was found to be closely associated with prevalence of HIV infection in most townships. Those in which it was not were distributed in A, N, and O counties (Figure 2). The male variable was found to be positively related to HIV infection in some northwestern counties (e.g. A and O counties) (*P*<0.05). However, this association was negative (*P*<0.05) or not statistically significant (*P*>0.05) in other counties (Figure 2). The drug rehabilitation variable was negatively related to HIV infection in most of the observed townships (*P*<0.05) (Figure 2).

### HCV infection and associated risk factors

**Figure 3:**
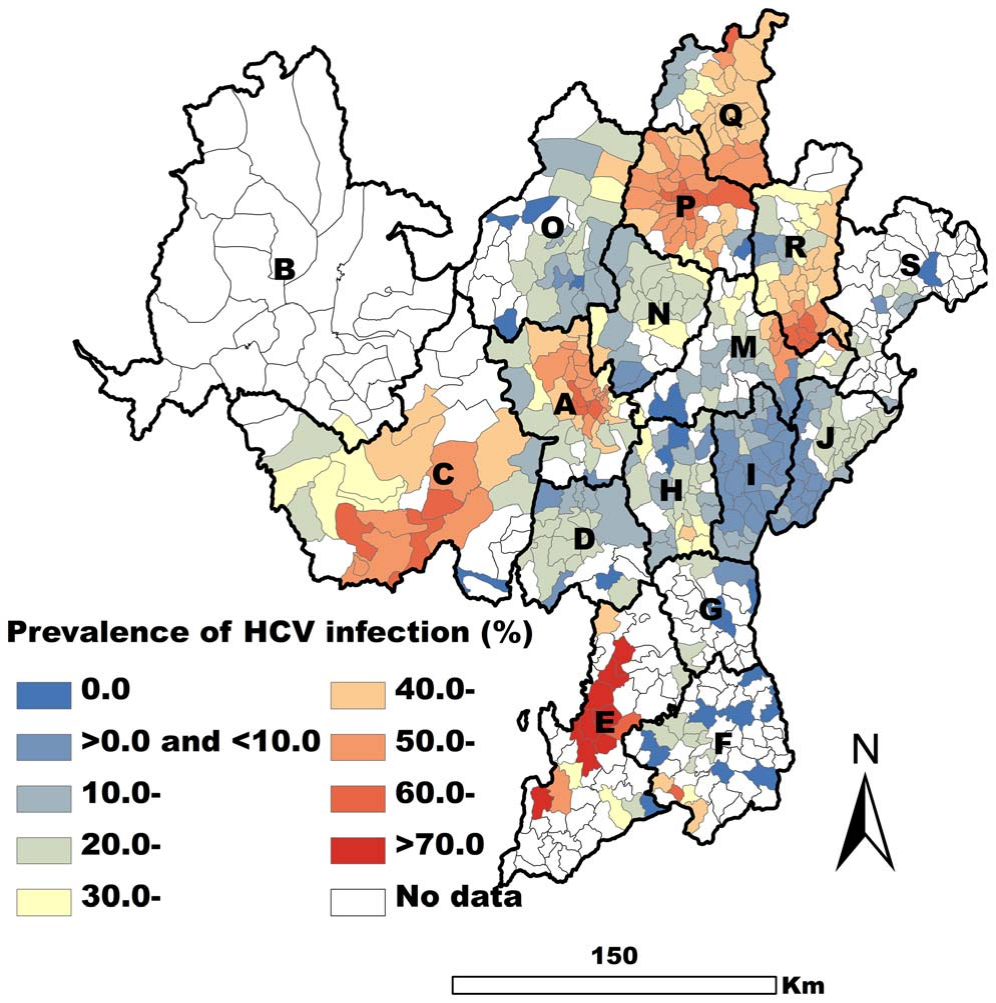
Spatial EB smoothed map of the prevalence of HCV infection by township.

A total of 5653 methadone patients were obtained for analysis of HCV infection, and the prevalence of HCV infection in these patients was 30.9% (1744/5653). They came from 384 of the 618 townships (Figure 3). For some patients tested for HIV were missed for HCV infection, fewer patients obtained for HCV analysis than HIV in our study.

The three variables (intravenous drug use, shared syringes, and drug rehabilitation) were found to be closely associated with the prevalence of HCV infection (*P*<0.01) in the OLS regression model. The fit of the OLS model was not good (AIC = −286.91, Adjusted R^2^ = 0.38), and the tolerance values indicated a little problem with multicollinearity (The multicollinearity condition number was 8.32). The low probabilities of the Jarque-Bera and White test scores indicated non-normal distribution of the error and the existence of heteroskedasticity (*P*<0.01). Moran's I (error) score was positive and highly significant (*P*<0.001) indicating a strong positive spatial autocorrelation of the residuals (Table 2).

**Table 2:** Association of the prevalence of HCV and risk factors.

	OLS				SLM				SEM			
Model fit												
R^2^	0.3840				0.7680				0.3447			
Adjusted R^2^	0.3791				0.4248				-			

Model estimation	Coefficient	t-statistic		*P*	Coefficient	Z-value		*P*	Coefficient	Z-value		*P*
Constant	0.1710	7.1605		0.0000	0.0295	1.1684		0.2427	0.1108	3.9386		0.0001
Rate of intravenous drug use	0.4600	7.4428		0.0000	0.2019	3.7611		0.0002	0.2303	3.3209		0.0009
Rate of sharing syringes	0.3606	5.5427		0.0000	0.1984	4.2039		0.0000	0.1944	2.1380		0.0325
Rate of participation in drug rehabilitation programs	0.1416	2.8433		0.0047	0.0573	1.6996		0.0892	0.3144	5.5162		0.0000
*ρ*/λ*	-	-		-	0.6335	7.002		0.0000	0.8484	32.7329		0.0000

Diagnostic tests	Value		*P*		Value		*P*		Value		*P*	
Normality of errors												
Jarque-Bera test	189.2884		0.0000						-		-	
Heteroskedasticity												
White test	98.9243		0.0000		-		-		-		-	
Spatial dependence												
Moran's I (error)	16.9804		0.0000		-		-		-		-	
Anselin-Kelejian Test	-		-		2.1301		0.1444		-		-	

*ρ: spatial lag term; λ: the error terms.

The results of the SLM regression model changed slightly, and all regression coefficients decreased, especially the drug rehabilitation variable. The regression coefficient of this variable ceased to show statistical significance (*P*>0.05) (Table 2). The ranking of the regression coefficients in the SLM regression model was the same as in the OLS model. The coefficient parameter (*ρ*) of the spatial lag term had a positive effect and was highly significant (*ρ* = 0.63, *P*<0.001), reflecting the spatial dependence inherent in the prevalence of HCV infection (Table 2). Allowing the spatial lag term to be spatially correlated not only improved the fit of the model but also eliminated the spatial effects (*P*>0.05).

As in the OLS model, the effects of the three variables remained positive and significant in the SEM model (*P*<0.05). However, the rank order of the regression coefficients changed, and the rank of the drug rehabilitation variable changed from third to first (Table 2).

In three regression models (OLS, SLM, SEM), the biggest change was observed in the regression coefficient of the drug rehabilitation variable. This variable was found to be significantly related to HCV infection in both the OLS and the SEM models, but the relationship was not statistically significant in the SLM. The drug rehabilitation variable ranked third in both the OLS and SLM models, but it ranked first in the SEM model (Table 2).

**Figure 4:**
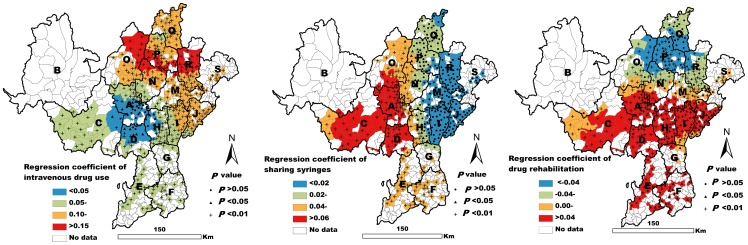
Spatial distribution of GWR coefficients of three variables associated with HCV infection.

The fit of the GWR model was high (AIC = −393.61, R^2^ = 0.56, adjusted R^2^ = 0.54). The GWR results showed intravenous drug use to be significantly associated with the prevalence of HCV infection in most of the townships studied here (*P*<0.05). This positive association was not found in only a few of the observed townships (*P*>0.05) (Figure 4). Sharing syringes was closely related to the prevalence of HCV infection in some western townships (*P*<0.05). However, this relationship was not statistically significant in some eastern townships (*P*>0.05) (Figure 4). The drug rehabilitation variable was positively related to the prevalence of HCV infection in some southern townships (*P*<0.05). However, this variable was negatively associated with HCV infection in some northern townships (*P*<0.05) (Figure 4).

### HIV/HCV co-infection and associated risk factors

**Figure 5:**
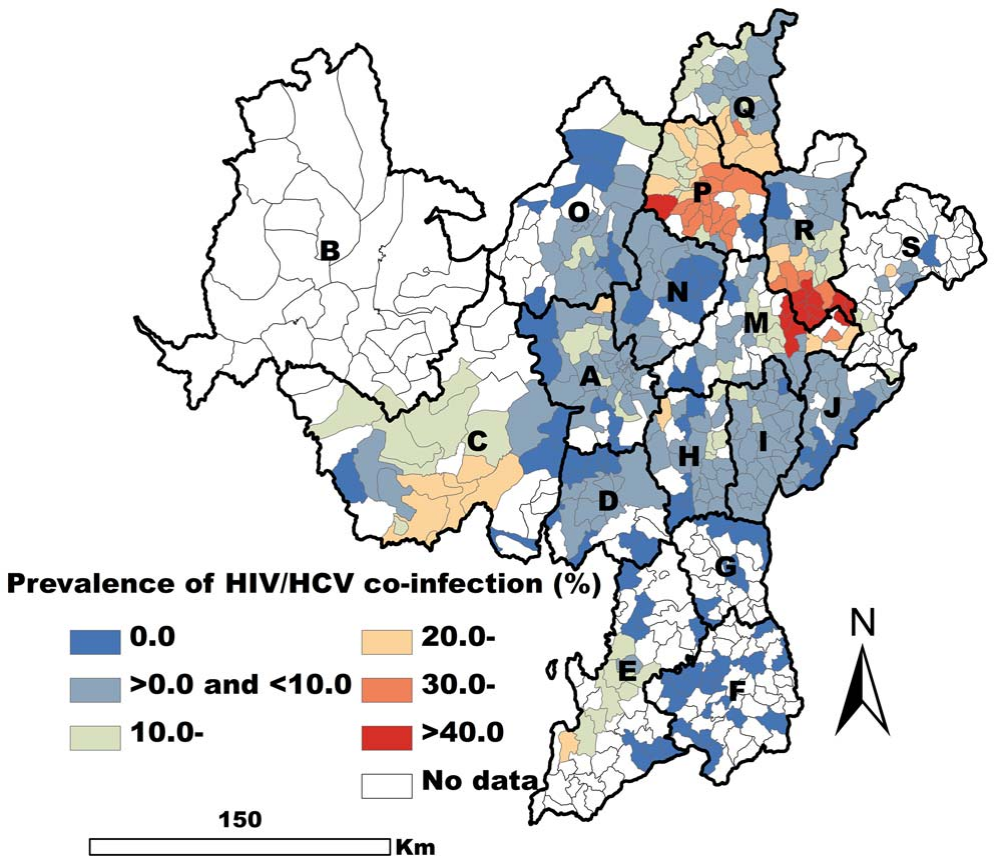
Spatial EB smoothed map of the prevalence of HIV/HCV co-infection by township.

A total of 5369 methadone patients were obtained for HIV/HCV co-infection analysis, and they came from the 381 of 618 townships. Some 11.0% (589/5369) of these patients were co-infected with both HIV and HCV (Figure 5).

Four variables were found to be closely related to HIV/HCV co-infection in the OLS regression model (*P*<0.001). Of these four variables, the effect of the male variable was negative, and that of other three variables remained positive. The fit of the OLS model was not good (AIC = −377.45, Adjusted R^2^ = 0.42), and the tolerance values indicated a slight problem with multicollinearity (The multicollinearity condition number was 17.56.) The results of the White test indicated the existence of heteroskedasticity (*P*<0.01). Moran's I (error) score was positive and highly significant (*P*<0.001) indicating strong positive spatial autocorrelation of the residuals (Table 3).

**Table 3:** Association of the prevalence of HIV/HCV co-infection and risk factors.

	OLS				SLM				SEM			
Model fit												
R^2^	0.4283				0.7846				0.3949			
Adjusted R^2^	0.4223				0.4638				-			

Model estimation	Coefficient	t-statistic		*P*	Coefficient	Z-value		*P*	Coefficient	Z-value		*P*
Constant	−0.0234	−0.5155		0.6065	−0.0246	−0.8614		0.3890	0.0045	0.1554		0.8765
Rate of intravenous drug use	0.4441	9.7181		0.0000	0.2379	5.7916		0.0000	0.3110	5.5899		0.0000
Rate of sharing syringes	0.6414	10.8225		0.0000	0.3153	5.2958		0.0000	0.3181	4.0729		0.0000
Relative number of men	-0.2511	-4.3361		0.0000	-0.1456	-3.6930		0.0002	-0.0572	-1.0819		0.2793
Relative number of Yi	0.2465	10.6072		0.0000	0.1175	5.0088		0.0002	0.1070	3.4837		0.0005
*ρ*/λ *	-	-		-	0.6021	7.0274		0.0000	0.8274	31.5600		0.0000

Diagnostic tests	Value		*P*		Value		*P*		Value		*P*	
Normality of errors												
Jarque-Bera test	4.3943		0.1111		-		-		-		-	
Heteroskedasticity												
White test	33.4550		0.0025		-		-		-		-	
Spatial dependence												
Moran's I (error)	17.8668		0.0000		-		-		-		-	
Anselin-Kelejian Test	-		-		3.3048		0.0691		-		-	

*ρ: spatial lag term; λ: the error terms.

As in the OLS model, the effects of the four variables remained significant (*P*<0.01) in the SLM model. The ranking of the regression coefficients did not change. Only the absolute values of the regression coefficients of all variables decreased (Table 3). The coefficient parameter (*ρ*) showed a positive effect and was highly significant (*ρ* = 0.60, *P*<0.001), indicating the spatial dependence inherent in the prevalence of HIV/HCV co-infection (Table 3). Inclusion of the spatial lag term not only improved the fit of the model but also eliminated the spatial effects.

As in the SLM model, the rank order of the regression coefficients remained unchanged the SEM model. However, the regression coefficient of the male variable no longer showed statistical significance (*P*>0.05) (Table 3). In the three regression models, the rank of the coefficients of the sharing syringes variable was always first (Table 3).

**Figure 6:**
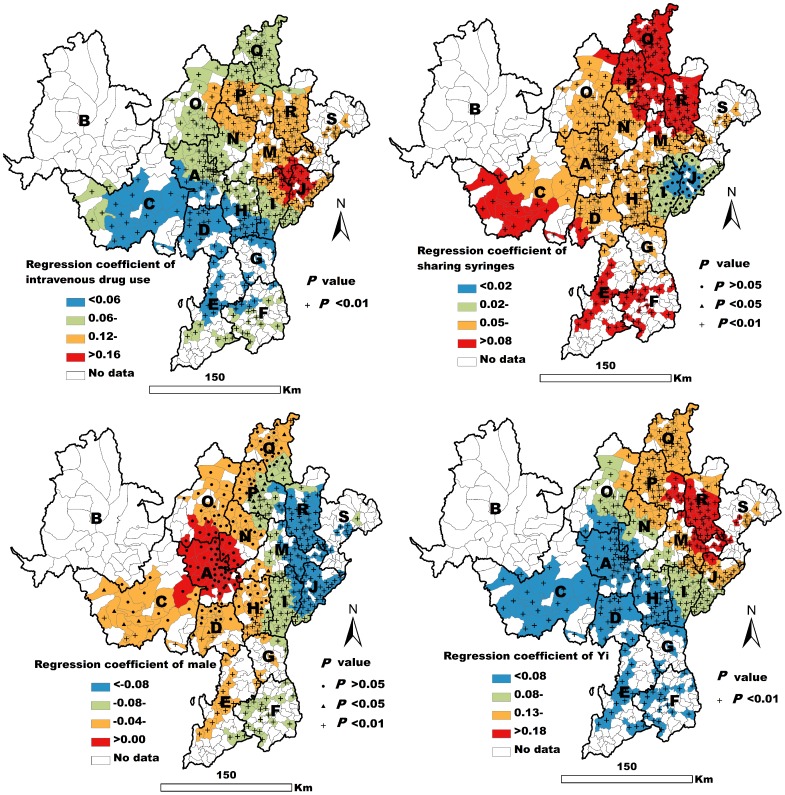
Spatial distribution of the GWR coefficients of four variables associated with HIV/HCV co-infection.

The fit of the GWR model was high (AIC = −501.81, R^2^ = 0.62, adjusted R^2^ = 0.59). The GWR results showed both intravenous drug use and Yi ethnicity to be closely related to the prevalence of HIV/HCV co-infection in all the townships observed in the present work (*P*<0.01) (Figure 6). Sharing syringes was closely associated with HIV/HCV co-infection in most of the observed townships (*P*<0.05). It was not closely associated with co-infection in a few of the townships in I, J, and M counties (Figure 6). The male variable was negatively related to HIV/HCV co-infection in some eastern and southwestern townships (*P*<0.05), but it was not statistically significantly related to co-infection in some northwestern townships (*P*>0.05) (Figure 6).

## Discussion

The present study makes a methodological contribution to field in that it integrates several spatial modeling techniques into the analysis of community characteristics associated with the prevalence of HIV and HCV infection at the population level. The present findings showed stark geographical variations in the prevalence of HIV infection, HCV infection, and HIV/HCV co-infection in the area studied. These observed spatial variations highlighted the clustering of HIV and HCV transmission across the Yi Autonomous Prefecture. The spatial lag parameters in the SLM models were all found to be statistically significant, suggesting that the risk of the two viral infections in a specific neighborhood was similar to the risk in surrounding neighborhoods. The absolute values of the regression coefficients of all variables except drug rehabilitation were lower in the SLM and SEM models than in the OLS regression model. The drug rehabilitation variable showed a higher value for HCV infection in SEM than in OLS, but the association was not found to be statistically significant in SEM model. This indicated that the coefficients of regression analyses may be overestimated if spatial autocorrelation is not taken into account. The present findings also suggest the existence of substantial and significant regional variations associated with some risk factors and these viral infections. For example, the male drug users in some northwestern counties had greater odds of being infected with HIV than female drug users, but the opposite was observed in some eastern counties. The drug rehabilitation variable was positively associated with the prevalence of HCV infection in some southern townships. However, a negative association was observed in some northern townships. This might explain why some associations between factors and these viral infections were judged differently in previous studies [13], [15]. In this way, these findings have critical implications for future research and clinical practice: spatial autocorrelation and regional differences should not be ignored in future studies of risk factors for these viral infections, and the makers of public health policy should give careful consideration to the substantial variation shown by the spread of HIV and HCV through populations and communities in different regions when formulating HIV or HCV control measures. This is especially important if one considers that there is no single global HIV or HCV epidemic. The Joint United Nations Program on HIV/AIDS has adopted the mantra “Know your epidemic; know your response” [9], [30], [31].

These analyses highlighted several important new insights into the correlations among HIV-, HCV-, and HIV/HCV co-infection. First, these findings showed several community characteristics to be associated with the prevalence of HIV infection, HCV infection, and HIV/HCV co-infection in the Yi prefecture. These findings suggested that the spatial variations in these viral infections could be explained to some extent by the characteristics of communities in which they took place. The three regression models (OLS, SLM, and SEM) all showed both intravenous drug use and sharing syringes to be positively related to the prevalence of infection with these two viruses in the area studied (*P*<0.01). These findings were consistent with a number of studies conducted at the individual level [13], [32], [33]. However, the values of the GWR coefficients of the two variables were different in different townships, though these similarities did not always show statistical significance. The present results showed that the standardized regression coefficients of sharing syringes were all greater than those of other variables for both HIV infection and HIV/HCV co-infection in OLS, SLM, and SEM models. These findings indicated that sharing syringes is the most important risk factor for both HIV infection and HIV/HCV co-infection in the area studied [34]. For HCV infection, the standardized regression coefficients of the sharing syringes variable were lower than those of the intravenous drug use variable in the OLS, SLM, and SEM models. This was consistent with a meta-analysis [13]. The GWR results suggested that injection drug use was closely associated with the prevalence of HCV infection in most of the observed townships, and sharing syringes was closely related to the prevalence of HCV infection in only half of the observed townships in western counties. These findings indicated that injection drug use might be the major risk factor for HCV transmission among drug users in the area studied. One plausible explanation for this is that HCV is highly transmissible, and it can spread easily through indirectly shared drug paraphernalia, such as cotton, cookers, and rinse water, even if drug users do not share syringes [35]–[37].

The present analysis showed that the rate of participation in drug rehabilitation programs was significantly negatively associated with the prevalence of HIV infection in most of the observed townships. This indicated that the drug rehabilitation performed in detoxification centers is probably an effective form of intervention and that it reduced the risk of HIV infection in the area studied [36]. One plausible explanation for this is that detoxification may increase drug users' knowledge of HIV and AIDS and the ways in which they are transmitted, allowing them to consciously reduce their engagement with risky behavior, especially sharing syringes [36], [37].The SLM model did not show drug rehabilitation to be associated with HCV infection. However, the SEM and OLS models both did. The GWR model did find drug rehabilitation to be significantly positively associated with the prevalence of HCV infection in some southern townships. However, it did not show this of some northern townships. This might explain why the results of the SLE, SEM, and OLS models were different. These findings indicate that there might be two different patterns of HCV transmission in the area studied. In southern townships, detoxification programs might increase the frequency of the sharing of cookers, cotton, rinse water, and drug solutions while reducing them in some northern townships [37]. Future studies should attempt to identify the factors that cause these differences.

Some demographic factors appeared to be important to the understanding of spatial variations in the prevalence of both HIV and HIV/HCV co-infection. Differences in the risk of both HIV infection and HIV/HCV co-infection were observed between sexes in the Yi prefecture. The male drug users in some northwestern counties had greater odds of being infected with HIV than female drug users, but the opposite was true in some eastern counties. These findings are similar to those reported by Zhuang et al. (2012) [13]. Like HIV infection, the male drug users in some eastern and southwestern townships had lower odds of being co-infected with HIV and HCV than female drug users, but the opposite was observed in some central counties. However, this difference was not found to be statistically significant. These findings might indicate that the transmission patterns of HIV infection and HIV/HCV co-infection are different in the different parts of the area studied. The first case of HIV was observed among drug users in eastern M county in 1995. From there, the HIV epidemic spread to all 16 counties and a city [38]. In general, male drug users were found to engage in more risky behavior than female drug users, such as sharing syringes. This increased their risk of HIV or HCV infection [13], [39]. In China, female drug users often have a higher risk of becoming infected with HIV or HCV through sex because a higher percentage of them participate in commercial sex work to sustain their drug use [39]. Condom use is infrequent in the prefecture [40]. One previous study indicated that the risk of infection from unprotected heterosexual intercourse was greater than that from sharing injecting equipment, especially for women [41]. The present findings indicate that intravenous drug use might be the dominant route of transmission in some northwestern counties, and heterosexual intercourse might be a major route of transmission route in some eastern counties. However, this needs further study. The three regression models (OLS, SLM, and SEM) showed that the Yi variable to be closely related to both the prevalence of HIV and of HIV/HCV co-infection, and the results of the GWR model also showed the Yi ethnic minority to be closely associated with prevalence of HIV in most of the townships examined and with the prevalence of HIV/HCV co-infection in all of them. These finding indicate that the Yi ethnic group is a high-risk population for both HIV infection and HIV/HCV co-infection. This might be related to the special cultural traditions of the Yi ethnic group. The Yi ethnic minority still maintains traditional social norms and values, including arranged marriage between individuals of the same social status and acceptance of casual sex. Condom use is infrequent among Yi [38], [40].

Despite their value, these findings have several limitations. One inherent limitation of any ecological study is that it is impossible to ascribe any observed relationship to individual respondents without risking an ecological fallacy [42]. However, for known risk factors like intravenous drug use, the present findings were consistent with a number of studies conducted at the individual level [13], [32], [33]. Other risk factors, like sexual behavior, were not considered here. Although the adjusted R^2^ of the regression models of HIV infection was high, especially the adjusted R^2^(0.81) of the GWR, and even though the factors included in the GWR model could explain 81% of the variance in HIV infection, the adjusted R^2^ of the regression models for HCV infection was not high. The factors included in the GWR model only could explain 54% of the variance in HCV infection. Inclusion of data on sexual behavior may have improved the fit of the models and should be explored in future studies. Only some drug users enroll in the national methadone clinics, and it is difficult to confirm whether methadone participants are a truly representative sample of the drug users in their community. However, it has been estimated the national HIV prevalence among the national methadone patients is not significantly different from that among drug users of non- national methadone clinics, as reported by national sentinel surveillance conducted from 2004–2009 [13].

## Conclusion

This is the first study to integrate several spatial modeling techniques for the examination of community characteristics associated with the prevalence of HIV and HCV infection at the population level. The present findings indicate that several community characteristics are associated with higher prevalence of HIV infection, HCV infection, and HIV/HCV co-infection. They also indicated the existence of substantial regional variations in these associations. The spatial modeling creates better representations of data such that public health interventions must focus on areas with high frequency of HIV/HCV to prevent further transmission of both HIV and HCV.
